# Tailored approach to participant recruitment and retention to maximize health equity in pediatric cancer research

**DOI:** 10.1186/s12874-024-02287-2

**Published:** 2024-07-24

**Authors:** Carolyn R. Bates, Renee M. Gilbert, Kelsey M. Dean, Keith J. August, Christie A. Befort, Shallyn Ward, Mary Gibson, Meredith L. Dreyer Gillette

**Affiliations:** 1 Department of Pediatrics, University of Kansas Medical Center, 2000 W Olathe Blvd, Mailstop, 4004, Kansas City, KS 66160 USA; 2 University of Kansas Cancer Center, Westwood, KS USA; 3 Department of Pediatrics, Children’s Mercy Kansas City, Kansas City, MO USA; 4 Department of Pediatrics, University of Missouri – Kansas City School of Medicine, Kansas City, MO USA; 5 Department of Population Health, University of Kansas Medical Center, Kansas City, KS USA

**Keywords:** Pediatrics, Oncology, Psychosocial, Recruitment, Retention, Diversity, Health equity

## Abstract

**Background:**

Lack of diversity in participants throughout the research process limits the generalizability of findings and may contribute to health disparities. There are unique challenges to recruitment of families to pediatric cancer research studies, especially for those from disadvantaged backgrounds. Thus, there is a need to evaluate the most effective recruitment and retention strategies to optimize equitable recruitment of diverse participants.

**Methods:**

The present study adapted and implemented methods outlined previously in the literature. These previous efforts were developed to address barriers to pediatric research, behavioral health intervention research and research with Black adolescents. Recruitment and retention strategies are described across four different time points: *pre-approach*,* initial connection*,* building connection* and *follow-up*. Eligible families of children with a pediatric cancer diagnosis were approached during a routine oncology visit. Once consented, enrollment and retention rates over three timepoints of data collection were recorded and evaluated.

**Results:**

Results indicated high rates of enrollment (86%) and retention (95%) for eligible participants. There were no trends in heightened attrition for any specific subgroup.

**Conclusions:**

The findings of this study are promising and suggest these recruitment and retention strategies may be useful in recruiting individuals from disadvantaged backgrounds.

## Background

Inequitable recruitment and retention of diverse groups to clinical research studies contributes to health disparities s [1, 2]. Despite National Institute of Health (NIH) initiatives to increase enrollment of participants from marginalized communities to federally-funded research, individuals from Black or African American, Latino/Hispanic, American Indian/Alaska Native, Asian and Pacific Islander backgrounds, those with low socioeconomic status and individuals from rural areas remain underrepresented [3–6]. Importantly, disparities in research participation occur at every stage of the research process – including observational studies, intervention development studies [2], basic and translational research, and psychosocial research [7, 8]. Lack of diversity in research participation limits the extent to which clinical scientists can develop and evaluate treatments that are generalizable to the population at large [4, 9]. Previously established barriers to research participation for racially and ethnic minoritized groups may include mistrust of institutions, researchers, and/or research agendas, uncertainty of short- and long-term outcomes of research participation, lack of access to information about research opportunities, fear of possible stigma, and concerns of health insurance discrimination [10].

Pediatric research studies may encounter unique barriers to equitable recruitment and participation of diverse participant groups [11] such as challenge of obtaining consent from both caregivers and children, in which children should be able to partake in developmentally appropriate, meaningful conversations about research participation [12]. However, caregivers often act as “proxy-decision makers” for their children in medical decision-making and research consent [11]. Caregivers report several concerns around allowing their child to participate in research [11]. One major concern is the potential medical risk to children in research participation. Caregivers also report psychological concerns and may view the research process as stressful or anxiety inducing [11, 13]. Further, caregivers report logistical burdens, including time commitment, excessive travel or financial constraints, and balancing competing tasks such as childcare or occupational demands [11]. All of these concerns may be heightened among caregivers from minoritized and underrepresented groups.

Within pediatrics, one population that is particularly challenging to recruit to clinical and psychosocial research is families of children with cancer [14]. A pediatric cancer diagnosis causes disruption to daily life; new and often demanding treatment regimens, frequent medical appointments, medication side effects and financial burden may hinder family capacity for research participation [15, 16]. Further, families from disadvantaged backgrounds may experience greater burden, including challenges such as difficulty navigating the medical system (e.g., for those with lower health literacy or those who do not speak English) or general medical mistrust which may lead to concerns about involving their child in research [11, 17]. Enrollment rates for psychosocial studies involving families of children with cancer range from 23 to 60% and attrition rates over a 1-year period range from 10 to 44% in prior studies [14, 18, 19]. In this high-risk population with relatively low disease incidence rates, low rates of enrollment present an issue for evaluating both impact and generalizability of treatments. Moreover, there is a paucity of research exploring how these rates vary across subgroups of participants who are typically underrepresented.

Experts have recommended several strategies to improve the recruitment and retention of underrepresented populations in pediatric research, including psychosocial studies of youth with cancer. In a qualitative study of caregivers of children with cancer, Canter and colleagues [20] suggested that recruitment efforts need to be flexible, repetitive and tailored to the individual family to promote research participation. The study also asserted the importance of research staff collaboration with the multidisciplinary care team. Similarly, a qualitative study by Kraft and colleagues [21] interviewed pediatric research staff to evaluate how they may build trusting research relationships with patients and families throughout the study process to facilitate participation in clinical research. Kraft and colleagues described the importance of a four-step recruitment and retention process: *pre-approach*,* initial connection*,* building connection* and *follow-up* and identifed key relationship building strategies during each step. Results highlighted a number of factors at the individual, relational and structural levels that may impact relationship development between staff and potential participants and their families, and proposed these are particularly important to recruit and retain participants from historically marginalized or under-represented groups. Finally, Ellis and colleagues [23] sought to understand effective outreach techniques to recruit Black adolescents and families into a behavioral health study. Findings suggested that persistent and flexible outreach efforts over an extended period of time were most effective in recruiting Black families [23]. Despite these well-documented recommendations to improve equity in pediatric research participation, few studies have documented recruitment or retention outcomes resulting from the use of these recommended methods. The goal of this paper is to (1) describe a programmatic approach to recruiting and retaining caregivers of youth with newly diagnosed pediatric cancer to a 1-year longitudinal psychosocial research study, with a focus on optimizing equitable recruitment of diverse participants, and (2) descriptively compare enrollment and retention rates across diverse groups based on child race, ethnicity, primary language, insurance (commercial vs. Medicaid/self-pay), and rurality.

## Methods

### Participants

The study took place at a cancer center within a children’s hospital in the Midwestern United States. Between 2021 and 2023, 257 families of children with a new diagnosis of cancer of any type were screened for recruitment to the study. The aim of the study was to evaluate changes in parenting during the first year of cancer treatment and the impact on child emotional and behavioral outcomes. Data collection as part of this study occurred over three time points; time 1 (T1) occurred 1–3 months after diagnosis, time 2 (T2) occurred 6–7 months after diagnosis, and time 3 (T3) occurred 12–13 months after diagnosis. Child eligibility criteria for the study included the following: age 2 to 14 years at time of study recruitment; receiving active cancer treatment at the time of recruitment (i.e., chemotherapy, radiation, or bone marrow transplant); lived at home with the participating caregiver at least 50% time. Caregiver eligibility criteria included being able to consent and complete surveys in English or Spanish. Children were ineligible if they were experiencing a recurrence, diagnosed with a second malignancy or if they were receiving palliative or non-curative treatment.

### Procedures

All study procedures, including informed consent from caregivers and assent from child participants 6 years of age and older, were approved through the Children’s Mercy Kansas City Institutional Review Board (STUDY00001654). Eligible families were approached to participate during a visit to the pediatric oncology clinic or inpatient unit. Recruitment and retention procedures are outlined in detail below. Families completed a series of questionnaires at three time points assessing psychosocial factors, including the Behavior Assessment for Children, Third Edition (BASC-3), [24] and the Psychosocial Assessment Tool (PAT 3.0) [25]. Electronic medical record abstraction was also conducted. Strategies for equitable recruitment and retention of families were adapted from Canter and colleagues [20], Kraft and colleagues [21], and Ellis and colleagues [22] and are documented in detail in Table 1. Below we summarize key aspects of each stage of the recruitment process.

**Table 1:** Recruitment and retention strategies, summarized

Kraft (2022) timeline steps	Canter (2020) & Ellis (2021) recruitment/retention recommendation	Associate strategies used in this study
Pre-Approach: *Consulting with the primary medical team prior to approaching the family is crucial to gauge their ability to participate in research (2022).*	1a: Individually tailored and multidisciplinary recruitment approach	• Presented study to primary oncology teams (oncologists, APRNs, and social workers) • Discussed how teams preferred to be contacted about recruitment (email) • Identified medical team champions • Established collaborative partnership with nursing and oncology social work during study visits
	1c: Timing of recruitment	• Always approached 4–16 weeks after initial diagnosis and treatment plan had been determined • Made regular contact with medical team to determine optimal recruitment timing • Extended recruitment window by 4 weeks (from 12–16 weeks) to provide additional flexibility
Initial Connection: *The research team should make efforts to connect with the family upon meeting for the first time (2022).*	1b: Presentation to parents is paramount	• Approached families in clinic to establish clear partnership with medical team • Adapted shorter verbal consent process after first several visits
	1d: Introduce study early and revisit recruitment often	• Offered individualized formats of recruitment: Initial contact in-person, follow-up in person (inpatient or outpatient) or by phone
	1e: Offer participation to each eligible caregiver	• Any eligible caregiver could participate based on family preference
Building Connection: *The relationship-building process is critical to recognize and accommodate the needs of families participating in research (2022).*	2a: Offer flexibility with scheduling and format	• Allowed follow-up survey completion at home or in clinic • Emailed surveys in advance of clinic appointments as a reminder • Met with caregivers at clinic visits or inpatient to offer surveys during down time
	2b: Reduce or eliminate common technological barriers	• Provided internet connected iPads during clinic visits and hospital admissions • 1:1 support for survey completion for families with literacy or language barriers • Offered hard copy alternatives to online surveys • Offered e-gift card or physical gift cards for compensation
	Other Strategies	• Provided escalated compensation across time points • Maintained consistent research assistant for each family to promote familiarity and relationship building • At weekly team meetings, reviewed all participants in window and problem-solved follow-up as needed
Follow Up: *Follow through with families is important in order to build longitudinal relationships (2022).*	2c: Provide psychosocial resources beyond study completion	• Designated “red flag” item process with alerts to patient social workers • List of hospital and community psychosocial resources given to every family

### Recruitment and retention procedures

#### Pre-approach

Before initiating data collection, the principal and co-investigators of the study met with pediatric oncology providers, including physicians, advanced practice registered nurse (APRNs), and oncology social workers to introduce the study and gather provider input. The medical teams expressed willingness to champion the study in the clinic by sharing brief information with patients and supporting research activities during routine clinic visits or inpatient admissions. Medical teams requested that the primary physician, APRN, and social worker for each pre-screened patient be notified through institutional email one week prior to approaching a patient in an upcoming clinic to facilitate mutual awareness and communication around any unique circumstances of the appointment. With the social work team, we discussed “red flag” items within PAT 3.0 measure [25] and our team’s IRB-approved plans for further safety assessment of any positively marked items. Social workers expressed willingness to partner with our team’s process for navigating red flags. After meeting with the primary oncology medical teams, study staff introduced themselves to other members of the oncology service, including oncology clinic and inpatient nurses. Finally, we posted flyers in provider-facing clinic areas that included photos of study staff and a brief overview of the study to promote awareness.

Leading up to clinic appointments, study staff emailed medical teams with the name and appointment date of pre-screened patients whom we planned to approach for study consent and/or follow-up survey completion. At times, medical teams responded and asked the study team to delay or refrain from approaching participants for research. In these circumstances, we responded to medical teams empathetically and kept in close contact. We reviewed the patients’ chart in the following weeks to gather information on their evolving clinical picture. We prioritized our relationships with the medical team and revisited participation over time. In some cases, we sent a brief reminder to the medical team that our study aims to capture a full range of experiences of families of children undergoing cancer treatment, and we also suggested that some families may find it meaningful or cathartic to share what they are going through during times of high stress. Using these methods, we were eventually able to obtain consent from all of the patients for whom medical teams asked us to delay our approach, except one patient, who we eventually determined was not able to be consented due to not living with their legal caregiver.

#### Initial connection

We prioritized meeting families in person, typically at regularly scheduled clinic appointments, and occasionally in the inpatient oncology unit, for the initial contact to introduce the study. Research assistants (RAs) identified the patient’s primary nurse for the day and re-introduced themselves, emphasizing that the RA’s intent was to work with the family’s availability that day and not interfere with the appointment. The RA also provided the nurse with an estimate of timing of various study components and noted that these components could be completed at separate times during the visit, or even on separate days if needed. This strategy allowed nurses to partner with the RAs and advise on optimal timing for approach and research activities.

When making the initial patient contact, RAs provided a brief overview of their role and purpose of visiting and asked the family if this was a good time for them to learn about the research study being conducted in the clinic. If the family stated that this was not a good day, RAs emphasized the study team’s flexibility and stated that we would return at a future appointment to introduce the study. Families were informed that they did not have to decide about whether to participate based on a brief encounter, and RAs were happy to return later to provide study information and determine the fit of this research for the family. If the family was willing to learn about the study during the initial encounter, RAs asked permission to take a seat in the room to provide additional information about the research.

#### Building connection

Informed consent to participate was obtained from all adult participants in the study. Children who were 6 years of age or older provided assent to study procedures. RAs provided study information and initially walked parents through a written consent process. However, after consenting 13 participants to the study, the team received patient feedback that the consent process seemed unnecessarily lengthy and arduous during clinic appointments. Thus, we sought permission from the IRB and were successful in adapting a shorter verbal consent process to reduce time and burden.

Following consent, RAs offered flexibility with scheduling and format for measurement completion. Options included completing surveys in clinic or at home with various accommodations available. Internet connected iPads were provided to participants during clinic visits or inpatient admissions. If participants elected to take surveys at home, the survey link was emailed, and participants were educated on how to complete surveys via their home computer or mobile phone. Paper forms were available as an alternative to online surveys for participants with vision impairment or participants who preferred not to use the internet for cultural or religious reasons (*N* = 2). During survey administration, RAs remained available for questions, and often engaged with the child or siblings to reduce demands on the caregiver. Participants were allowed to skip any questions that they did not want to answer. Finally, survey compensation was available through an e-gift card or physical gift card depending on participant preference.

Our study team made several additional adaptations for health equity in the building connection phase. For participants with limited literacy, RAs read survey items aloud and recorded participant response (*N* = 2). For example, one Spanish-speaking caregiver had difficulty understanding the Spanish-translated questionnaire items even when they were read to her by the RA. The participant requested to call her spouse for assistance. The RA then read both caregivers the items in Spanish and allowed them to discuss the question in their own words before providing an answer for the study. This participant later shared that she was concerned about answering questions “wrong” and thus felt more comfortable with her husband assisting her participation. This adapted approach resulted in a significant participant burden, however, as the questionnaires took this family 6 total hours to complete, including 3 visits with the RA, in comparison to 20–30 min for most other participants. This family declined to complete repeated measures surveys at T2 and T3, however they were agreeable to remaining in the study for medical record abstraction.

#### Follow-up

To minimize attrition (i.e., reduction in number of participants engaged throughout study), each participant was assigned to a consistent RA—typically the RA who initially consented the participant to the study—to promote familiarity and relationship building. RAs took field notes at each patient time point to keep a log of family-specific needs or preferences surrounding measurement completion. We offered flexible methods for follow-up survey completion and provided prompts to patients via email and phone when they were eligible to complete T2 and T3 surveys; we capped our remote contacts at one email and voicemail per week. We also obtained IRB approval to reach out to participants via the EMR’s patient portal but did not find this to be necessary during the study. Approximately 54.7% of families completed surveys remotely without additional in-person follow-ups needed. Remote methods of survey completion were well-suited for participants who lived far away from the hospital, including several from remote rural areas. RAs visited participants at follow-up clinic appointments or inpatient admissions to provide gentle reminders around survey completion and to problem-solve any issues accessing the surveys (e.g., some participants struggled to launch surveys on their phone at follow-up time points). RAs also reminded participants about escalated compensation at each time point ($25, $50, and $75 respectively), which several participants stated was motivating. For participants who had not claimed or used their gift cards from T1, RAs problem-solved for family’s access to their gift cards at T2 and T3 time points. Problem-solving to ensure gift card access was particularly important for families with fewer resources (e.g., inconsistent access to phone or internet).

### Analytic plan

Data were analyzed using IBM SPSS Statistics (Version 27). All data were assessed for missing variables. For all participants screening out of the study, the team screening log notes were assessed and frequencies were calculated to capture reasons for ineligibility. Among families who were eligible for the study, descriptive analyses including means, standard deviation, and percentiles were conducted to describe demographic characteristics for participants who (a) enrolled in the study, (b) declined to participate, and (c) had incomplete measures at T2 or T3. Representativeness of the participating sample was evaluated by visually contrasting trends in demographic characteristics between participating and non-participating families.

## Results

### Participant recruitment and demographics

**Fig. 1:**
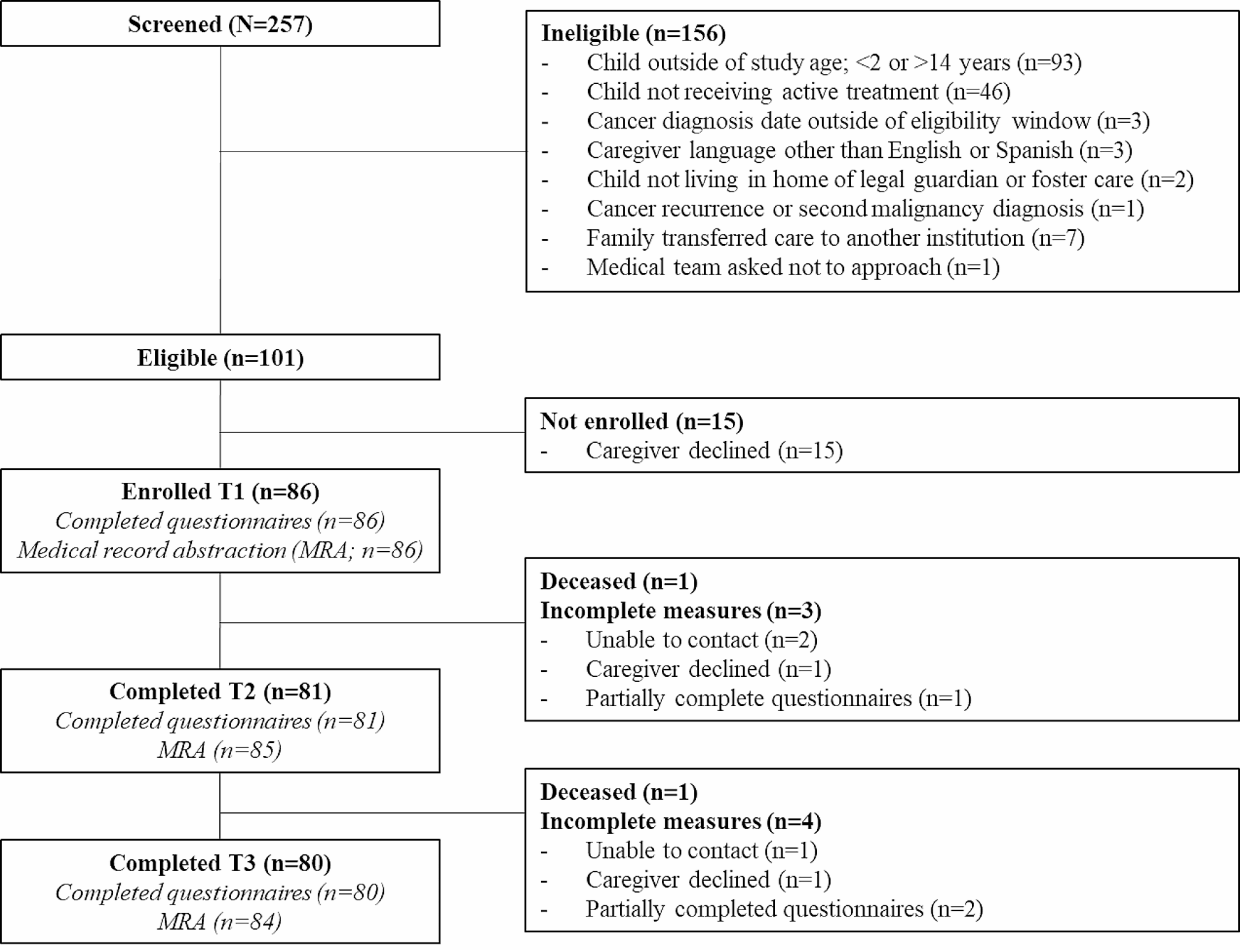
Consort diagram of participant recruitment and retention from screening through study completion at Time 3. *Note* T1 = Time 1; T2 = Time 2; T3 = Time 3, study completion

A comprehensive summary of participant screening, eligibility, recruitment, and retention can be found in Fig. 1. Of the 257 families screened, 156 families were deemed ineligible based on study inclusion criteria, with age as the most common reason for screening out of the study and child not receiving active treatment (e.g., surveillance or palliative care) as the second most common reason for screening out. All 101 families who met eligibility criteria were approached for study participation at an in-person clinic visit or admission to the inpatient unit. Fifteen caregivers declined participation, resulting in 86 total enrolled participants.

Demographics of enrolled participants are included in Table 2. Mean age of participating children was 7.8 years (SD = 3.9 years; Median age = 7 years, Modal age = 3 years). Child sex was 52% male, 48% female. Participating caregivers were 88% (*n* = 76) women. Demographics are largely representative of the broader population of children served by our Cancer Center, which draws from a large Midwestern catchment area including Missouri, Kansas, and surrounding states. Consistent with the population in this area, our participants were majority White and Non-Hispanic. However, all Spanish-speaking participants (*n* = 10; 11.6%) whom we approached for this study were enrolled.

**Table 2:** Demographics of enrolled participants, eligible participants who declined enrollment, and participants with incomplete measures

	Enrolled	Declined participation	Incomplete measures
N	86	15	6
Mean age (y)	7.8	7.0	10.1
Child sex (n)			
Male	44	6	2
Female	42	9	4
Child race (n)			
White/Caucasian	53	11	3
Black / African American	8	3	1
American Indian / Alaska Native	1	0	1
Other	7	0	0
Multiracial	12	1	1
Asian	4	0	0
Hawaiian / Other Pacific Islander	1	0	0
Child ethnicity (n)			
Hispanic	22	0	2
Non-Hispanic	64	15	4
Caregiver primary language (n)			
English	76	15	4
Spanish	10	0	2
Child insurance (n)			
Commercial	46	Unknown*	2
Medicaid/Self Pay	40	Unknown*	4
Family zip code rurality (n)			
Urban	69	Unknown*	5
Large rural	8	Unknown*	0
Small rural	6	Unknown*	0
Isolated rural	3	Unknown*	1

*Unknown data was not collected during screening

### Retention

Figure 1 documents retention of participants throughout the study. All enrolled participants completed T1 measures, including caregiver-reported questionnaires and medical record abstraction by the study team. Between T1 and T2, one participating child died of their cancer, resulting in 85 eligible participants at T2. All measures were completed by 81 of the 85 eligible participants at T2. Between T2 and T3, a second participating child died of their cancer, resulting in 84 eligible participants at T3. All measures were completed by 80 of the 84 eligible participants at T3. At both T2 and T3, the study team conducted medical record abstraction for all eligible families, even if caregiver-report questionnaires were incomplete. Although one participating family denied completing caregiver-reported questionnaires at T2 and T3, this participant consented to ongoing medical record abstraction. No participants asked to be removed from the study altogether.

Only 6 participants in the study were not retained through T3, with two participants who became deceased during the study. Demographics of participant attrition are included in Table 2. Reasons for participant non-completion are summarized in Fig. 1.

## Discussion

The aim of this study was to describe a programmatic approach to recruiting and retaining caregivers of youth with newly diagnosed pediatric cancer to a 1-year longitudinal psychosocial research study, with a focus on optimizing equitable recruitment of diverse participants. Results indicated that a flexible approach to recruitment has potential to maximize participation and retention of diverse groups to longitudinal pediatric psycho-oncology research. Results also support the literature on the importance of ongoing relationship building with the primary medical teams to tailor study team approaches to individual participant needs.

Our study demonstrated a strong rate of enrollment (86%) in the context of a limited number of eligible participants. Other psychosocial studies approaching families in the first several months after diagnosis have obtained enrollment rates ranging from 20 to 60% of eligible participants [14, 18, 19]. Given that our research occurred at a single site serving patients 0–18, study inclusion criteria resulted in most participants being screened out due to age or type of treatment. Thus, a high enrollment rate was essential to meet recruitment goals. Results suggest that a personalized, flexible approach to recruitment may maximize likelihood of participant enrollment in the pediatric cancer context. There has been limited work evaluating the effort (i.e., workforce costs) required to implement these flexible recruitment strategies consistently in various populations, or whether they are cost effective for larger trials. On one hand, such strategies certainly require more recruitment effort per patient than other methods (e.g., opt-out letters) [26]. However, high rates of enrollment and retention suggest that this effort could be cost-effective, especially among clinical populations with lower incidence rates or those who are historically underrepresented in research. Future studies may examine cost-effectiveness of tailored recruitment strategies in various study designs and populations.

It is also notable that in comparison to other psychosocial studies in pediatric oncology, our study used a longer window of open enrollment at T1 (i.e., 4–16 weeks after diagnosis in comparison to Stehl and colleagues [14] approaching within the first 2 weeks). However, in comparison to other studies, our window for T1 participation was much shorter (e.g., Canter and colleagues [18] approached within 1 year of diagnosis). While longer enrollment periods may result in greater heterogeneity of T1 timing, it may also allow staff to flex and tailor recruitment strategies based on family needs at the start of treatment. This may be valuable when there are a limited number of eligible participants for a given study.

Representative samples in formative research studies are essential to capture the full range of patient experiences [27]. Our enrollment and retention outcomes are largely representative of the patients served at our institution and Cancer Center, including patients from rural areas and those with government-sponsored insurance coverage (i.e., Medicaid). Despite having a broad catchment area, our site has lower racial and ethnic diversity compared to other areas of the country, so replication of these strategies is needed to confirm the fit of recruitment strategies in other communities. However, our team used strategies from Ellis and colleagues [22] and others [20, 21] in different areas of the country (e.g., Delaware, Detroit and Seattle) that have more diversity. It is promising that all Spanish-speaking caregivers whom we approached for participation enrolled in the study, suggesting that if given the opportunity and resources (e.g., Spanish-speaking study staff and appropriately translated measures) this population is willing to participate in research. Efforts to remove burden of research likely contributed to participant sample. However, persistent constraints of the study measurements, including not being able to sufficiently adapt measures to overcome burden for participants with limited literacy, highlight areas for ongoing consideration and improvement.

This study demonstrated generally strong retention of enrolled participants compared to studies in similar samples (e.g., range of attrition from 10 to 44%) [14, 18, 19], though it is notable that these were intervention studies in comparison to our observational study. We did not find trends in heightened attrition from any specific subgroup, which was promising. However, our examinations of within-group attrition were limited given that we had such high retention rates. Ongoing relationships with medical teams allowed us to tailor our team’s approach and follow-up to specific families and situations. While we appreciated opportunities to follow-up with families in person during clinic appointments, it is also notable that a sizeable subsample of families completed follow-up measures without additional in-person reminders. This resulted in follow-up data collection being less time intensive for research staff, in comparison to initial recruitment and consenting.

The purpose of this study was to describe a programmatic, tailored approach to participant recruitment and retention in a sample of caregivers of youth with a new diagnosis of cancer using published recommendations for pediatric populations. Strengths of the study include the flexible integration of published methods alongside individual participants needs to maximize participation from diverse groups within our Cancer Center, including children from rural areas, those with Medicaid, and those from Spanish-speaking families. The generalizability of our findings is limited by our single site of data collection. Moreover, while our study did not include an intervention, future directions for research could include use of these methods for researchers conducting clinical trials given the similar site and clinical nature of data collection.

## Conclusion

The present study found strong enrollment and retention rates for participants of all demographic subgroups, showing promising effectiveness for the outlined recruitment and retention strategies. Achieving equitable recruitment of individuals from underrepresented backgrounds in pediatric research is crucial to address health disparities. Thus, future research should continue to implement and evaluate these methods in order to recruit and retain families from underserved backgrounds.

## Data Availability

No datasets were generated or analysed during the current study.
